# Editorial: Food and immunity: tackling the diseases of the 21st century

**DOI:** 10.3389/fnut.2025.1656710

**Published:** 2025-07-21

**Authors:** Ujjawal Sharma, Bunty Sharma

**Affiliations:** ^1^Department of Human Genetics and Molecular Medicine, Central University of Punjab, Bhatinda, India; ^2^Department of Biotechnology, Graphic Era (Deemed to be University), Dehradun, Uttarakhand, India

**Keywords:** nutrition, oxidative stress, immunity, editorial, translational potential

Scientific understanding of how diet, gut microbiota, oxidative stress, and immune function interact to influence health has significantly grown over the past 10 years. Recent studies on topics ranging from pediatric health to livestock nutrition and even edible vaccines show that nutritional science, microbiome research, and immunology are increasingly connected. This editorial summarizes key findings from nine studies, offering a clear view of the current state and future research opportunities in human and animal health.

A recent review of the interaction between gut microbiota indicates that diet has a multivariate, bidirectional influence on the structure of the microbiome as well as gut function (1). Shang et al. emphasized the significance of precision nutrition interventions in the promotion of microbial balance and wellbeing. Low-FODMAP diets, individualized probiotics, and even fecal microbiota transplantation are exemplary examples of such interventions (Shang et al.). These findings are in agreement with larger-scale initiatives toward the personalization of dietary recommendations based on the individual microbial and metabolic profiles. This new trend is likely to transform the management of gastrointestinal and metabolic disease (2).

Among the higher-order themes that are conveyed is that of redox homeostasis. Lupu et al. observe that an imbalance between reactive oxygen species and antioxidants is a pathophysiological mechanism in child obesity with impacts on both cardiovascular and metabolic risk. This was supported by previous biomarker research, which showed that obese children experienced decreased glutathione activity with increased lipid peroxidation (3). Two studies using the NHANES database present strong evidence for the protective effect of dietary antioxidants against oxidative stress (4). One of these studies detected a large negative correlation between niacin levels and the incidence of stroke and investigated dietary niacin and stroke risk in adult Americans between 1999 and 2018. Increased intake was seen to reduce the risk of stroke, especially in those with risk factors such as obesity and hypertension. Another NHANES study in 2009–2014 investigated the relationship between periodontitis and the composite dietary antioxidant index. This study detected a direct relationship between nutrition and oral immune modulation, with diets containing high levels of antioxidants such as zinc, vitamin C, vitamin E, and selenium being linked with a reduced prevalence of periodontal inflammation (Meng et al.; Qiu et al.).

Today's studies on probiotics, prebiotics, and postbiotics explore the therapeutic function of diet ingredients regulating gut health in greater detail. Such agents were referred to by the review authors as a “therapeutic symphony” that may coordinate the gut's and immune system's interactions, especially in autoimmune and inflammatory disorders. The review suggests that they work in concert: probiotics enable the re-colonization of healthy microbial strains, prebiotics stimulate their growth, and postbiotics, like microbial metabolites like short-chain fatty acids, modulate immune cell functions. As a package, they are a powerful, non-drug strategy for promoting mucosal integrity and restoring balanced immune responses.

The relevance of the findings goes beyond the domain of human health. Promising results are presented in animal trials on immune modulation with the support of nutritional supplements. Supplementation of diets with vitamins, rumen-protected amino acids, and trace minerals has improved bovine immune capacity, as well as antioxidant and anti-inflammatory processes, especially during the periparturient phase when the risk of mastitis is elevated, as illustrated in a study in dairy cows. These findings emphasize the overlap of immune-nutritional processes across species and indicate that identical methodologies used in human health can be transferred to optimize nutritional interventions in animals.

In widening the scope of investigation, another study investigated the possible effect of dietary cannabinoids and hemp on the health and performance of different animal species. Although the regulatory and mechanistic base is still under development, studies show that cannabinoids, particularly CBD, modulate gut barrier function, inflammatory reactions, and metabolic efficacy in animals. However, there lies the promise of improved agricultural productivity and the development of cannabinoid-derived therapeutic interventions for humans on the basis of the use of plant-derived modulators that influence metabolism and the immune system.

Edible vaccine manufacture is a new method of nutrition-mediated health intervention. A recent milestone study on TOMAVAC, an oral COVID-19 vaccine produced in tomatoes, found that mice and humans generated neutralizing IgG antibodies after eating such genetically engineered tomatoes. This is a cutting-edge convergence of immunology with plant biotechnology and nutritional delivery systems. Edible vaccines can provide the solution to global vaccine problems through reduced reliance on the cold chain and increased availability in resource-poor environments.

Individualized diets are becoming increasingly popular in the context of immunologically mediated disease in children. A study showed that food-specific IgG4-targeted elimination diets decreased considerably symptoms of allergy in children. The findings are in favor of the hypothesis that long-term exposure to food antigens may precipitate immune activation and symptomatology in a subgroup of children with a specific category of pediatric patient, even though there remains some controversy regarding IgG4 testing as a part of allergy diagnosis. The finding contributes to the increasing volume of literature supporting the use of precision diets in the management of inflammatory and allergic disease.

The overlap of these studies highlights shared central themes: that oxidative stress and inflammation are prevalent in many chronic diseases; that diet and manipulation of the microbiome are an attractive, non-surgical therapeutic strategy; and that interdisciplinary, nutrition-focused approaches can enhance the health outcomes in animals and humans. Furthermore, these studies recognize the necessity for a deeper understanding of immune modulation and molecular nutrition to inform the development of plant-based immunotherapies, enhance productivity in livestock, and address childhood obesity.

It is clear that the implications of these results must be accepted in future research. Future research must aim at the integration of multi-omics technologies into clinical and agricultural research, such as metabolomics, redox proteomics, and microbiomics. The translational value of these results could be greatly increased by randomized controlled trials assessing oral microbiome-directed treatments for periodontitis and antioxidant-supplemented nutrition in children. Novel functional foods, such as edible vaccines and cannabinoid-derived animal supplements, also have the potential to be enabled by new safety and regulation paradigms.

Finally, the converging evidence reinforces a new paradigm of agriculture and medicine that views the gut as a key axis of health, prioritizes the quality of diet and antioxidant balance, and employs food as preventive and therapeutic medicine. Today, it is common for nutritional science, microbial ecology, immunology, and biotechnology to intersect. This inter-disciplinary research provides actionable and scalable solutions to some of the biggest health issues of the time.
